# Production and validation of a good manufacturing practice grade human fibroblast line for supporting human embryonic stem cell derivation and culture

**DOI:** 10.1186/scrt103

**Published:** 2012-03-28

**Authors:** Nilendran Prathalingam, Linda Ferguson, Lesley Young, Georg Lietz, Rachel Oldershaw, Lyn Healy, Albert Craig, Helen Lister, Rakesh Binaykia, Radhika Sheth, Alison Murdoch, Mary Herbert

**Affiliations:** 1 NorthEast England Stem Cell Institute, Centre for Life, Times Square, Newcastle upon Tyne NE1 4EP, UK; 2 Institute for Ageing and Health, Newcastle University, Centre for Life, Times Square, Newcastle upon Tyne NE1 4EP, UK; 3 Institute for Cellular Medicine, Centre for Life, Times Square, Newcastle upon Tyne NE1 4EP, UK; 4 UK Stem Cell Bank, National Institute for Biological Standards and Control, Blanche Lane, South Mimms Potters Bar, Hertfordshire, EN6 3QG, UK; 5 School of Agriculture, Food and Rural Development, University of Newcastle, Kings Road, Newcastle upon Tyne NE1 7RU, UK; 6 Institute for Genetic Medicine, Newcastle University, Central Parkway, Times Square, Newcastle upon Tyne, NE1 4EP, UK; 7 Newcastle Fertility Centre, Centre for Life, Times Square, Newcastle upon Tyne NE1 4EP, UK

## Abstract

**Introduction:**

The development of reproducible methods for deriving human embryonic stem cell (hESC) lines in compliance with good manufacturing practice (GMP) is essential for the development of hESC-based therapies. Although significant progress has been made toward the development of chemically defined conditions for the maintenance and differentiation of hESCs, efficient derivation of new hESCs requires the use of fibroblast feeder cells. However, GMP-grade feeder cell lines validated for hESC derivation are not readily available.

**Methods:**

We derived a fibroblast cell line (NclFed1A) from human foreskin in compliance with GMP standards. Consent was obtained to use the cells for the production of hESCs and to generate induced pluripotent stem cells (iPSCs). We compared the line with a variety of other cell lines for its ability to support derivation and self-renewal of hESCs.

**Results:**

NclFed1A supports efficient rates (33%) of hESC colony formation after explantation of the inner cell mass (ICM) of human blastocysts. This compared favorably with two mouse embryonic fibroblast (MEF) cell lines. NclFed1A also compared favorably with commercially available foreskin fibroblasts and MEFs in promoting proliferation and pluripotency of a number of existing and widely used hESCs. The ability of NclFed1A to maintain self-renewal remained undiminished for up to 28 population doublings from the master cell bank.

**Conclusions:**

The human fibroblast line Ncl1Fed1A, produced in compliance with GMP standards and qualified for derivation and maintenance of hESCs, is a useful resource for the advancement of progress toward hESC-based therapies in regenerative medicine.

## Introduction

Progress in the use of human embryonic stem cells (hESCs) derivatives for cellular therapies will require the production of clinical-grade lines under the control of good manufacturing practice (GMP) [1]. The ultimate goal is to increase reproducibility in the production of hESCs by developing chemically defined culture conditions by using recombinant proteins for hESC derivation and culture. A major hurdle is to dispense with the use of feeder cells, which are conventionally used to promote and support hESC self-renewal [2]. However, the ability of feeder-free culture systems to maintain genetic stability remains controversial [3]. Furthermore, reproducible techniques for deriving new GMP-grade hESC lines from human blastocysts without the use of feeder cells remain to be developed [4]. To date, only a single report describes successful hESC derivation in the absence of feeder cells [5]. Interestingly, the two lines derived under these conditions acquired karyotypic abnormalities during subsequent culture [5]. Thus, in the absence of a chemically defined, GMP-compliant method for efficient derivation of hESCs, the production of new clinical-grade hESC lines will require a supply of GMP-grade feeder cells.

The use of fibroblast cells as feeder cells for derivation and long-term culture of hESCs has been well documented [2,6-8]. Although the majority of currently available hESC lines were derived on MEFs, concerns about animal pathogens and immunogens in cells destined for human therapy [4] motivated scientists to explore the use of human fibroblasts [6,9-13]. Several reports have indicated that human fibroblasts originating from fetal, neonatal, and adult skin are capable of supporting self-renewal of established hESCs [6,9-13]. However, not all human fibroblast cell lines are equally supportive of hESC self-renewal [12]. Transcriptome analysis of supportive and nonsupportive fibroblast cell lines identified a panel of differentially expressed proteins, including extracellular matrix proteins and growth factors, thought to be supportive of hESC self-renewal [14].

A number of studies have reported on the use of human fibroblast feeder cells for derivation of new hESC lines [7,10,12,15-18], but the field currently lacks ready access to a GMP-compliant human feeder cell line validated for this purpose. Furthermore, when we embarked on the derivation of GMP-grade hESCs, we were unable to source fibroblasts that had been produced to GMP and characterized for hESC derivation and culture. Here we describe the production, characterization, and validation of a GMP-grade fibroblast line derived from human foreskin with specific ethics approval and consent for hESC derivation and culture.

## Materials and methods

### Regulation and compliance

This study was approved by the Local Research Ethics Committee (Sunderland Research Ethics Committee) and was licensed by the UK Human Fertilisation and Embryology Authority. Blastocysts were obtained after informed donor consent. Human foreskins were obtained after parental consent. The premises for the production of the clinical-grade fibroblast line has been licensed by the UK Human Tissue Authority (HTA) for testing, processing, storage, distribution, and import/export of human tissue (HTA license number 22111). All processes associated with the derivation, expansion, and cryopreservation of the master cell bank (MCB) of NclFed1A were carried out in accordance with the Newcastle University Biomanufacturing Facility Quality Management System (QMS), which operates in accordance with appropriate legislation, guidance, and regulation published by the Medicines and Healthcare products Regulatory Agency (MHRA) and the HTA. All documentation related to the QMS and to the production process was created and managed by using Q-Pulse software (Gael Ltd, UK.

### Derivation and expansion of human fibroblasts

We derived human foreskin fibroblasts from tissue obtained from donors deemed to be of low risk based on their medical history. This included healthy children of ~6 months of age undergoing circumcision for religious reasons with no known infection or disease. Because no vertical transmission of prions has been documented in humans, the use of tissue from a young child minimizes the risk of prion contamination [19]. All human tissue was transferred to the processing laboratory in PBS. The tissue was dissociated with a scalpel (VWR, UK) and incubated with Collagenase Type IV (Invitrogen, USA Cat. No. 17104-019) at 37°C for 40 minutes. Samples were washed by centrifugation and plated in a T25 or T75 flask (TPP; Switzerland) with either FBS growth medium (DMEM (Invitrogen, Cat. No. 11995-065), 10% FBS (Invitrogen, Cat. No. 10099-141) and 1 × glutamine (Invitrogen, Cat. No. 25030) supplemented with 1× Pen/Strep (Invitrogen)) or with xeno-free hESC medium; KOSR-XF (KO DMEM (Invitrogen), 15% KnockOut Serum Replacement-XenoFree (Invitrogen), 0.1 m*M *NEAA (Invitrogen); 0.1 m*M *β-mercaptoethanol; 2 m*M *Glutamax; 8 ng/ml FGF2 (Invitrogen)). Cells were incubated at 37°C and 5% CO_2_, medium was changed every 48 to 72 hours, and cells were passaged when cells were confluent. Cells were deemed confluent when the growth surface of the flask was covered by cells (Additional file 1, Figure S1).

For passaging, the culture medium was removed from flasks and replaced with Tryple Select (Invitrogen); the fibroblasts were incubated for 5 minutes at 37°C. The cells were washed by centrifugation and passaged. Cells cultured from the pre-seed bank (PSB) to the master cell bank (MCB) were cultured in FBS growth medium without penicillin and streptomycin. Cells were passaged at a ratio of 1:6 in T75, T150, or T300 flasks with an estimated plating density of 3.5 × 10^4 ^cells/cm^2^). When confluent at P5, the MCB was cryopreserved; the choice of flask was based on the maximum number of flasks an operator could handle in one session.

### Cryopreservation of fibroblasts

Fibroblasts were dissociated with Tryple Select, washed by centrifugation, and resuspended in freeze medium (10% DMSO (Sigma, UK) and 90% FBS (Invitrogen)). The resuspended cells were aliquoted into 1-ml aliquots in 2-ml cryovials (TPP) that were cooled by using controlled-rate freezing (Mr Frosty; Nalgene, USA) at 1°C/min. After cryopreservation, cell counts and viability were carried out by using a Vi-Cell (Beckman Coulter, USA).

### Inactivation of fibroblasts

Fibroblasts were inactivated by using either mitomycin C or X-ray irradiation. For mitomyocin C inactivation, fibroblasts were incubated with 10 μg/ml of mitomyocin C for 2.5 hours at 37°C and 5% CO_2_. The mitomyocin C was washed out by using FBS growth medium, and washing was repeated 7 times. Samples were incubated overnight in the FBS growth medium and cryopreserved, as described previously. For X-ray inactivation, fibroblasts were exposed to 50 Gy (Faxitron, USA). Samples were incubated overnight and cryopreserved.

### Source of human blastocysts

Embryos used to determine the efficiency of hESC derivation were donated by couples undergoing assisted-conception treatment. Embryos were produced *in vitro *by conventional oocyte insemination or by intracytoplasmic sperm injection (ICSI) and cultured in G1 medium (Vitrolife, Sweden) for 2 to 3 days until the best-quality embryos were selected for transfer to the uterus or for cryopreservation. The embryos used in this study were cryopreserved but were no longer required for treatment. Cryopreservation and thawing was performed by using a Vitrolife Freeze medium (Vitrolife) and Thaw medium (Vitrolife). Thawed embryos were cultured in G2 medium (Vitrolife) for 3 to 4 days until they developed to the blastocyst stage. All blastocysts, regardless of quality, were included in the study and were randomly allocated to explantation on three different feeder cell lines.

### hESC stem cell derivation and culture

hESC derivations were carried out in a fully enclosed isolator cabinet (Vitrosafe). Human blastocysts were dissociated by using two insulin needles (Becton Dickinson, USA); the inner cell mass (ICM) was removed and plated on Cellstart (Invitrogen) with either inactivated human foreskin fibroblasts (NclFed1A) or MEFs. Samples were incubated in hESC medium; KOSR (KO DMEM (Invitrogen), 20% KnockOut Serum Replacement (Invitrogen), 0.1 m*M *nonessential amino acids NEAA (Invitrogen); 0.1 m*M *β-mercaptoethanol; 2 m*M *Glutamax; 8 ng/ml FGF2 (Invitrogen)) supplemented with 5% Quinns Advantage Protein Supplement (Rochford Medical, Ltd, UK). The plated ICMs were incubated for 3 days at 37°C, 5%CO_2_, and 5%O_2_, and were checked daily for the presence of outgrowths. Initial hESC colonies were dissected by using insulin needles and passaged on to fresh feeder cells in medium that was changed every 2 to 3 days. For enzymic passaging, once the cells became 65% to 85% confluent, they were washed once in PBS medium and then incubated for 5 to 15 minutes in Tryple Select (Invitrogen). They were washed once with centrifugation in hESC medium and passaged at a ratio of 1:3 or 1:6.

### PCR analysis

RNA extraction was carried out by using Dynabeads mRNA Direct (Invitrogen) as described in the user manual, and cDNA was synthesized by using Superscript III (Invitrogen). The PCR primers are described in Additional file 2, Table S1. PCR reactions were carried out by adding Biomix red (Bioline, UK), as described in the user manual. Conditions for the PCR were 94°C for 2 minutes, 30 × (94°C for 30 seconds, 58°C for 30 seconds, 72°C for 30 seconds), and 72°C for 15 minutes. PCR products were run on a 1% agarose gel.

### Population doubling time

NclFed1A was thawed and seeded in T25 flasks. After incubation for 48 hours, cell counts were determined by using a Vi-Cell. This was repeated at 24-hour intervals for 4 days. Counts were repeated 3 times for each sample.

### Estimation of the number of cells in a confluent flask

NclFed1A was passaged into 8 × T150 flasks; when deemed confluent (Additional file 2, Figure S1), the cells were dissociated by using Tryple Express (Invitrogen), resuspened, and three cell counts were measured for each flask by using a Vi-Cell. The number of cells in a confluent flask and per square centimeter was then determined (Additional file 2, Figure S1).

### Immunostaining

hESC colonies or fibroblasts were grown on coverslips. The cells were fixed in 4% PFA, blocked with a 10% wt/vol milk-powder solution for 1 hour at room temperature and incubated overnight at 4°C with an anti-NANOG human polyclonal antibody raised in goat (R and D Systems, USA), an anti-OCT4 polyclonal antibody raised in rabbit (Abcam, UK), HFF-Cellect (Cellartis, Sweden), or 5B5 (Abcam). The cells were washed and incubated with either Alexa Fluor 488 donkey anti-goat immunoglobulin (Molecular Probes, USA), Alexa Fluor 555 donkey anti-rabbit immunoglobulin (Molecular Probes), or Alexa Fluor 488 donkey anti-mouse immunoglobulin (Molecular Probes). Nuclear staining was carried out by using Draq5 (Biostatus, UK). Samples were washed 3 times before imaging. Samples were imaged by using an inverted confocal microscope (Zeiss, Germany).

### Flow-cytometry analysis for cell-surface markers

FACS analysis was carried out as described [20]. In brief, cells were dissociated with Tryple and incubated for 1 hour with the fibroblast-specific marker HFF-Cellect (Cellartis), and an antibody raised against either Tra-1-60, Tra-1-81, Tra-2-54, SSEA3, or SSEA4. Cells were washed and incubated for 30 minutes with the appropriate secondary antibody. Analysis was carried out by using a flow cytometer (Becton Dickinson).

### Determination of Neu5GC concentrations

Cells were treated with trifluoracetic acid at 80°C for 1 hour and derivatized by using DMB solution (7 m*M *1,2-diamino-4,5-methylene dioxybenzene (Sigma); 1.4 *M *acetic acid (Sigma), 0.75 *M *β-mercaptoethanol (Sigma), and 18 m*M *sodium hydrosulfite (Sigma)) for 2 hours 30 minutes at 50°C and run on an HPLC (Dionex) by using a C8 column (Agilent AD-LC-139, USA). Samples were run at 0.90 ml/min in an isocratic solution of 7% methanol, 9% acetic acid, and 84% water. Neu5Gc detection was carried out by using a fluorescence detector (Dionex, UK) with an excitation wavelength of 373 nm and emission wavelength of 448 nm.

### Karyotyping and genotyping

Karyotyping and genotyping were contracted to The Doctors Laboratory, which is accredited by the National External Quality Assessment Service (NEQAS; UK).

### Statistical analysis

Analysis of variance was used to investigate the effect of the fibroblast cell line or the passage number of fibroblasts on the expression of pluripotency markers in hESCs. Χ^2 ^analysis was used to compare fibroblast cell lines for hESC derivation efficiency. All data are expressed as mean ± SD. As it is envisaged that NclFed1A will be used only as feeder cells derived from the MCB, therefore all population-doubling data (PD) are expressed from the MCB.

## Results

### Selection of culture medium

In view of immunogenic and pathogenic considerations, our starting point was to perform preliminary experiments to determine whether hESCs could be derived in culture media free of animal products (xeno-free media). A foreskin sample, obtained from a child being treated for hypospadias, was divided in two sections and seeded in a T75 flask with either FBS growth medium or KOSR-XF, which was the only GMP-compliant xeno-free medium available at the time. Tissue seeded in the FBS growth medium attached within 24 hours and fibroblasts were observed 7 days after seeding; the cells became confluent 5 days later. By contrast, cells seeded in the KOSR-XF medium did not attach, and fibroblast cells were not observed. In a separate experiment, we found that coating of culture dishes with Cellstart promoted attachment of foreskin tissue in the presence of KOSR-XF; however, it took 28 days for the cells to become confluent. After passaging of the primary culture at a ratio of 1:3, the cells took 14 days to become confluent. Because of the slow doubling time (estimated to be 112 hours, based on passage time, compared with 26.7 hours for cells grown on FBS) and poor morphology of the cells, we opted to use qualified FBS-containing medium for subsequent culture. We selected GMP-compliant FBS from Australia, which was screened for adventitious agents, cell-plating efficiency, and chemical and physical properties. However, cells cultured in FBS have been found to contain the nonhuman form of sialic acid [21], which is immunogenic for humans [22]. Our strategy for minimizing this is described later.

### Production of a GMP-grade fibroblast line

**Figure 1:**
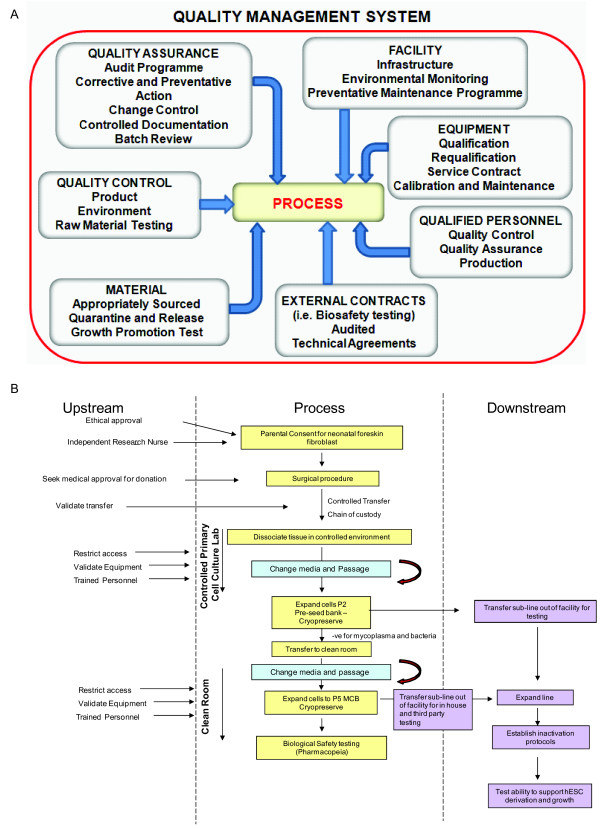
**Derivation of the clinical grade fibroblast line NclFed1A**. **(a) **A detailed description of the Quality Management System (QMS) required for processing cells to good manufacturing practice (GMP; passaging of NclFed1A). **(b) **Schematic showing the general processes involved in deriving a fibroblast cell line under the control of GMP. "Upstream" includes all processes that are in place before the derivation and expansion of the fibroblast line. "Downstream" refers to biosafety and functionality testing of the line.

We next established a GMP-compliant process for the derivation and production of clinical-grade fibroblasts in accordance with the Biomanufacturing Facility Quality Management System (QMS; Figure 1a). This included generating 37 standard operating procedures (SOPs) and 32 record forms to cover all procedures and equipment used in this cell line-production process. In accordance with the QMS policies and procedures, we documented all staff training, audits, and supplier approvals. A fully documented quarantine and release procedure for materials used in the productions process was implemented by the QM team. A complaints procedure with the associated Corrective and Preventive Actions (CAPA) was also implemented by the QM team.

Fibroblast tissue for GMP-compliant production was obtained from a healthy 6-month-old child undergoing circumcision for religious reasons. After excision, the foreskin tissue was transferred from the operating theater to a QM-controlled primary cell-culture laboratory through a documented chain of custody (Figure 1b). The tissue sample was split into two sections (CPR/HGP/Ncl/Fed1a and CPR/HGP/Ncl/Fed1b), and each section was seeded into a T75 flask. Attachment and outgrowth of cells with fibroblast morphology were observed by day 4. Both sublines became confluent within14 days of seeding and were cultured to P1 or P2 for production of a pre-seed bank (PSB) consisting of eight vials of each subline. After microbial and mycoplasma clearance, and validation for hESC culture and derivation (see later), the subline CPR/HGP/Ncl/Fed1A (NclFed1A) was transferred to the Grade B clean-room suite for onward culture.

To establish a master cell bank (MCB) of NclFed1A, cells from the PSB were seeded at an estimated density of 3.5 × 10^4 ^cells/cm^2 ^and grown to confluence before being passaged at an equivalent density until P5 (equivalent to eight population doublings (PDs) from the PSB), when they were frozen to form an MCB consisting of 200 vials containing 7.5 × 10^6 ^cells/vial. Seven vials were used to test for bacterial and fungal contaminants, mycoplasma, and retroviruses by using Eu Pharmacopoeia methods (Table 1). In view of the large quantity of cells required for viral testing, an additional two vials of the MCB were passaged to produce 1.9 × 10^8 ^cells, which were cryopreserved and tested for bovine viruses (according to 9CFR guidelines (Section 113.46, 113.47 and 113.53)), and for human viruses (HIV1 and 2 provirus, HTLV1 and 2 provirus, HAV, HBV, HCV, HHV6, HHV7, HHV8, hCMV, EBV, SV40, and B19).

**Table 1:** Biosafety tests carried out on the NclFed1A master cell bank

Test	Reference	Test result
Sterility test and qualification of test article by direct inoculation method	[38]	Negative

Test for *Mycoplasma *spp.(Including mycoplasmastasis assay)	[38]	Negative

Retroviruses: detection of reverse transcriptase (F-Pert assay)	[39-47]	Negative

Detection of human viral pathogens (HIV 1 and 2 provirus, HTLV 1 and 2 provirus, HAV, HBV, HCV, HHV-6, HHV-7, HHV-8, hCMV, EBV, SV40 and B19) by real-time polymerase chain reaction (PCR).	[48]	Negative

Detection of bovine viruses with in vitro adventitious assay according to 9CFR Guidelines	[43,45,47,49-52]	Negative

### Characterization of NclFed1A as a fibroblast cell line

**Figure 2:**
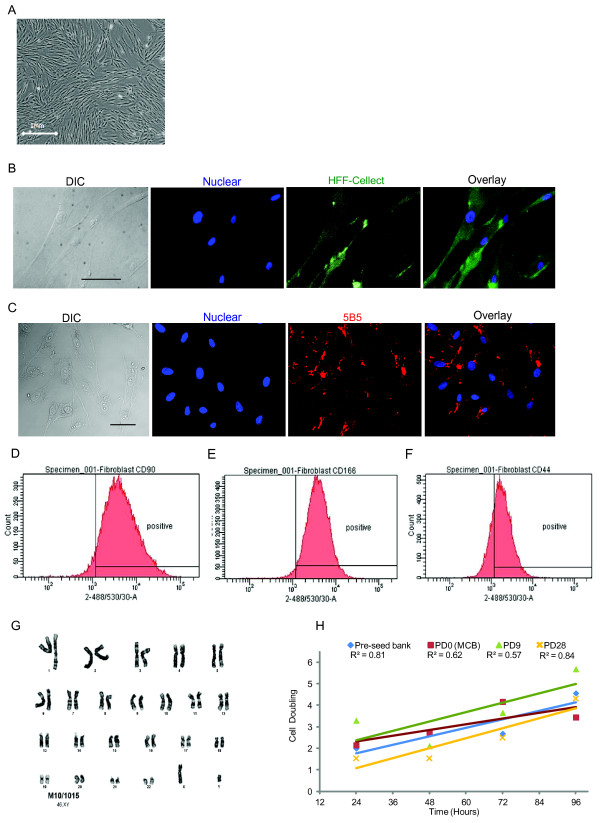
**Characterization of NclFed1A as fibroblasts**. **(a) **NclFed1A has a normal fibroblast morphology. **(b) **Cells were positive for HFF-Cellect, a mouse monoclonal antibody derived from mice immunized with human foreskin fibroblasts (scale bar, 50 μm). **(c) **Cells were positive for the fibroblast marker 5B5 required for triple-helix formation in collagen (scale bar, 50 μm). **(d-f) **Histograms from flow cytometry showing the proportion of cells positive for **(d) **CD90, **(e) **CD166, and **(f) **CD44. **(g) **NclFed1A had a normal male karyotype. **(h) **The doubling time of NclFed1A was 26.7 hours; no significant difference was found between PD0, PSB, MCB, and 10 (PD10) and 28 (PD28) cell doublings from the MCB.

NclFed1A had a typical fibroblast morphology (Figure 2a) and stained positive for HFF-Cellect (Figure 2b), a mouse monoclonal antibody derived from mice immunized with human foreskin fibroblasts. In addition, immunofluorescence labeling showed positive staining for 5B5 (Figure 2c), a fibroblast-specific protein required for triple-helix formation in collagen [23]. Moreover, flow-cytometry analysis showed that NclFed1A was positive for proteins previously found to be expressed in fibroblasts, including CD90 [24-26]; CD166 [25], and CD44 [27] (Figure 2d through 2f).

For the purposes of traceability, we determined the DNA profile of the cell line by using 15 polymorphic autosomal DNA markers (Additional file 2, Table S2). Karyoptype analysis of the MCB showed a normal male karyotyped 46XY (Figure 2g). Further to characterize the cell line and to assess the time to confluence during routine culture, we measured the doubling time of NclFed1A across several passages. A mean doubling time of 26.7 ± 13 hours was calculated for cells thawed from the PSB, the MCB, 10 population doublings from the MCB (PD10), and 28 population doublings from the MCB (PD28). No significant difference in doubling time was found between samples (Figure 2h). Subsequent observations indicated that cell senescence did not become apparent until 54 population doublings from the MCB.

### NclFed1A supports hESC derivation and culture

As an initial assessment, we tested NclFed1A for expression of genes predicted to be important for the maintenance of hESC pluripotency [14]. By using PCR analysis, we tested NclFed1A at the PSB, MCB, PD10, and PD28 for expression of transcripts encoding extracellular matrix proteins Col1A1, Col3A1, Col5A1, fibronectin 1, heparin sulfate proteoglycan, and hyaluron synthase 2 (HAS2), as well as the ligands and growth factors FGF2, IGFBP3, ADAM33, and Grem1. With the exception of Col5A1 and ADAM33, we found that all factors were expressed at PSB, MCB, and PD10 (Table 2). By contrast, at PD28, only Col1A, HAS2, IGFBP3, and Grem1 were detected.

**Table 2:** Presence (✓) and absence (x) of transcripts in NclFed1A at PSB, MCB, PD10, and PD28 that have been predicted to support hESC culture

NclFed1A	Col1A1	Col3A1	Col5A1	FN1	HSPG2	HAS2	IGFBP3	FGF2	ADAM33	Grem1
PSB	✓	✓	X	✓	✓	✓	✓	✓	X	✓

MCB	✓	✓	X	✓	✓	✓	✓	✓	X	✓

PD10	✓	✓	X	✓	✓	✓	✓	✓	X	✓

PD28	✓	X	X	X	X	✓	✓	X	X	✓

**Figure 3:**
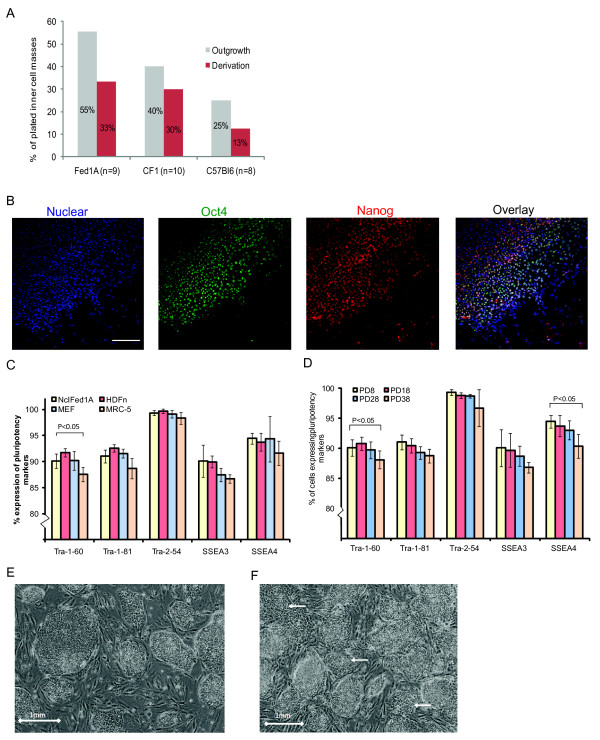
**NclFed1A supports human embryonic stem cell (hESC) derivation and culture**. **(a) **The proportion of outgrowths and hESC lines produced by using NclFed1A and MEFS (derived from CF1 and C57Bl6 strains) as feeder cells. **(b) **hESCs cultured on NclFed1A for 24 passages were positive for the pluripotency markers NANOG and OCT4 (scale bar, 100 μm). **(c) **A greater proportion of cells expressed Tra-1-60 when cultured on NclFed1A than on MRC-5 (*P *< 0.05); no significant difference was noted between the expression of pluripotency markers after culture on NclFed1A, iHDFn, and iMEF. **(d) **The expression of pluripotency markers was similar after culture on NclFed1A at PD8, PD18, and PD28; at PD38, fewer cells expressed Tra-1-81 and SSEA4 (*P *< 0.05). **(e) **hESCs cultured on NclFed1A at P15 had normal hESC morphology. **(f) **hESCs cultured on NclFed1A at PD38 showed signs of differentiation (arrows).

We next tested the ability of NclFed1A to support hESC derivation from blastocysts. The efficiency of derivation was compared with MEFs from CF1 and C57BL6 mouse strains; both of which have been reported to be supportive of hESC derivation and/or culture [28-30]. After explantation of the ICM onto the inactivated fibroblasts, the proportion of outgrowths for NclFed1A was similar to that observed on MEFs (five of nine (NclFed1A) versus four of 10 (CF1) and two of eight (C57Bl6)). Subsequently, hESC colonies were observed on all fibroblast lines between days 6 and 11 (three of nine (NclFed1A) versus three of 10 (CF1) and one of eight (C57Bl6)) (Figure 3a). To test the ability of NclFed1A to support the proliferation and pluripotency of hESCs, two of the lines derived (Ncl12 and Ncl(R)14) on NclFed1A were cultured to P24. Both cell lines retained normal hESC morphology and, at P24, expressed OCT4 and NANOG (Figure 3b).

Further qualification of NclFed1A for use a feeder cell line for hESCs was conducted by the UK Stem Cell Bank (UKSCB) as follows. The hESC lines Shef1, RH-1, HUES-9, and NCL5 were cultured in parallel on inactivated MEFs (MEF), neonatal foreskin fibroblasts (HDFn), fetal lung fibroblast (MRC-5), and NclFed1A. After five passages, the percentage of cells expressing the pluripotent surface markers was determined with flow cytometry. No difference was found in the proportion of cells expressing Tra-1-60, Tra-1-81, Tra-2-54, SSEA3, and SSEA4 between NclFed1A, MEFs, and HDFn; however, a greater proportion of hESCs cultured on NclFed1A expressed Tra-1-60 than did those on MRC-5 (*P *< 0.05; Figure 3c and Additional file 2, Table S3). No significant differences were noted between NclFed1A at PD8 to PD28 in the proportion of hESCs expressing the pluripotent surface markers Tra1-60, Tra1-81, Tra-2-54, SSEA3, and SSEA4 after five passages (Figure 3d and Additional file 2, Table S4). However, by PD38, a significant decline was apparent in the proportion of hESC cells expressing Tra-1-81 and SSEA4 compared with hESCs cultured on feeders at PD8 (*P *< 0.05; Figure 3e, f, and Additional file 2, Table S4). Consistent with this, hESCs cultured on NclFed1A at PD38 showed morphologic signs of differentiation (Figure 3e and 3f). Thus, NclFed1A remains supportive of hESC self-renewal up to PD28. This is surprising, given that expression of the hESC supportive factors was greatly reduced at an equivalent number of population doublings (Table 2).

### Neu5Gc content is reduced after culture in xeno-free medium

**Figure 4:**
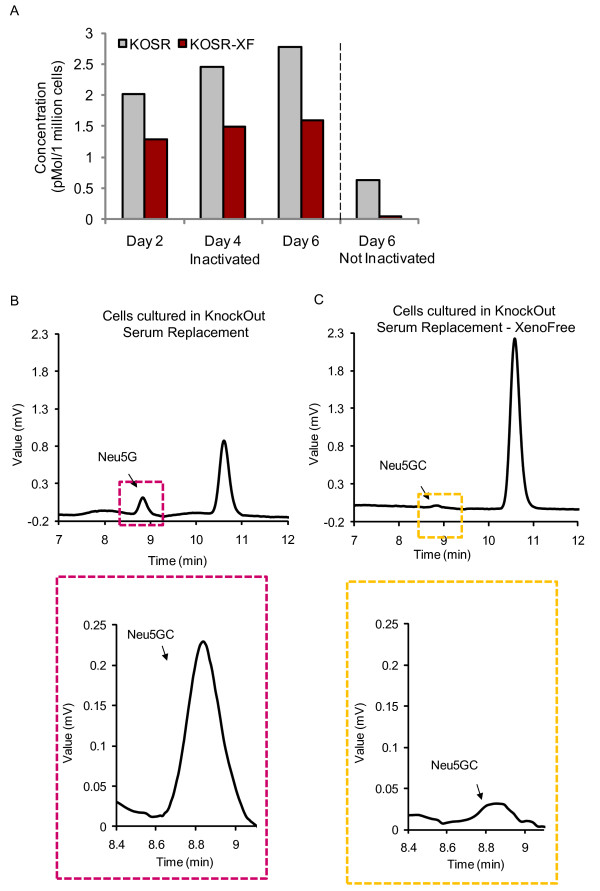
**High-performance liquid chromatography (HPLC) analysis showing the concentration of Neu5Gc in cells cultured in xeno-free human embryonic stem cell (hESC) medium (KOSR-XF) and xeno-containing medium (KOSR)**. **(a) **The concentration of Neu5Gc in inactivated and noninactivated NclFed1A cells cultured in KOSR-XF and KOSR medium for 2 to 6 days. **(b, c) **The HPLC trace of Neu5Gc concentration in noninactivated NclFed1A cultured for 6 days in **(b) **KOSR and **(c) **KOSR-XF.

Given that cells cultured in the presence of animal-derived products have been reported to contain the nonhuman form of sialic acid (Neu5Gc), which is immunogenic for humans [31], we determined whether the sialic acid content of NclFed1A could be reduced by transferring the cells to a xeno-free medium (KOSR-XF) before using them as feeder cells for hESCs. The concentration of Neu5Gc was measured with HPLC at 2-day intervals for 6 days after transfer of inactivated NclFed1A cells into KOSR-XF medium. By day 2, a 64% reduction in Neu5Gc content was found, compared with inactivated fibroblasts cultured in xeno-containing hESC (KOSR) medium at day 2 (Figure 4a). No significant further reduction was observed for 6 days.

We then tested whether the level of Neu5Gc could be further reduced by culturing NclFed1A in KOSR-XF medium for 6 days before inactivation. This strategy resulted in a 93% reduction in Neu5Gc concentration (0.63 versus 0.04 pMol/1 million cells; Figure 4b and 4c) compared with cells grown in xeno-containing KOSR medium. Even though the fibroblasts proliferated slowly in KOSR-XF, this did not affect their ability to support self-renewal of hESCs after inactivation (data not shown).

## Discussion

Here we report on the successful production of a GMP-grade fibroblast line (NclFed1A), characterized for use as feeder cells for the derivation and long-term culture of hESCs, as summarised in Table 3. The efficiency of hESC derivation from human blastocysts was comparable to that of MEF lines. In addition, NclFed1A compared favorably with commercially available fibroblasts in supporting proliferation and pluripotency of a number of widely used hESC lines. To the best of our knowledge, this is the first report that describes the production of a GMP-grade fibroblast line characterized for the derivation and culture of hESCs.

**Table 3:** The characteristics of NclFed1A

Characteristics	Result
Characterization of NclFed1A	
	
Expression of fibroblast markers	Expresses HFFCellect, 5B5, CD90, CD44, and CD160
Karyology	Normal male karyotype
Identification	STR analysis determined
Cell-doubling time	26.7 ± 13 hours
Biosafety testing on the MCB	
	
Sterility	Not detected
Mycoplasma	Not detected
Retroviruses	Not detected
Human viruses HIV 1 and 2 provirus, HTLV 1 and 2 provirus, HAV, HBV, HCV, HHV-6, HHV-7, HHV-8, hCMV, EBV, SV40, and B19	Not detected
Bovine viruses (9CFR)	Not detected
Ability to support hESCs	
	
Ability to support hESC derivation	33% (three of nine blastocysts plated)
Ability to support hESC culture	Yes (up to 28 population doublings from the MCB)

Our tissue of choice was foreskin fibroblasts, as it is easily accessible from healthy individuals undergoing circumcisions for religious practices. Furthermore, as the tissue was sourced from a child of about 6 months old, it is in compliance with the US Food and Drug Administration (FDA) regulations relating to the risk of prion contamination of primary tissue sourced from donors in the UK born before 1996 [32]. As further precautionary measures to minimize the transmission of infectious agents, we selected healthy low-risk donors based on their medical histories. Further preventive measures may include donor-blood biosafety testing that is routinely carried out for organ transplants and when tissue/cells are immediately transferred to a recipient. Because NclFed1A is intended to support the proliferation of hESCs and not for direct therapeutic use, we confined our biosafety testing to the cell line.

Although the use of xeno-containing media is not incompatible with GMP compliance, culturing in xeno-containing media results in increased sialic acid (Neu5Gc) concentration and the risk of immune rejection of hESC derivatives [31]. However, our attempts to derive a feeder cell line in the absence of animal components were hampered by the lack of a suitable xeno-free GMP-grade culture medium. We therefore opted to use FBS-containing medium, which has previously been used to produce cells intended for therapeutic use. For example, FBS-containing media have been used for several years for keratinocyte-based human therapies [33]. More recently, clinical grade hESC lines have been produced by using xeno-containing medium [15]. To minimize the risks associated with FBS, we used qualified FBS sourced from Australia that has had no reported cases of BSE, and tested the cell line for pathogens and retroviruses in accordance with Eu Pharmacopoeia. These results were negative, so we tested whether a reduction in immunogenicity could be achieved after transfer of the feeder cells into xeno-free medium. In agreement with previous studies, we found that the concentration of Neu5Gc was greatly reduced by culturing cells in KOSR-XF medium [31,34]. A more substantial effect (93% reduction) was achieved when cells were transferred to the KOSR-XF medium before inactivation.

Tests for mRNA expression of proteins thought to be supportive of self-renewal [14] revealed that eight of the 10 transcripts predicted to support hESCs [14] were present up to PD10 from the MCB. By contrast, only four (Col1a, HAS2, IGFBP3, and Grem1) were detected at PD28. This indicates that the gene-expression profile changes during the repeated passage of dermal fibroblasts derived from primary tissue. Surprisingly, this did not have any detectable effect on the ability of NclFed1A to support hESC self-renewal. This implies that feeder cell-derived Col3A1, Col5A1, ADAM33, FGF2, fibronectin, and heparin sulfate proteoglycan is not essential for maintenance of hESC lines, by using our culture conditions.

Finally, although significant progress has been made in the production of defined matrices such as laminin [35,36], vitronectin [37], and fibronectin [35] to support self-renewal of hESCs, a reliable feeder-free system for hESC derivation has not yet been reported. Thus, in the short term, at least, production of new clinical grade hESC lines will require a source of GMP-compliant feeder cells validated for this purpose. Importantly, specific consent was obtained for the use of NclFed1A as a feeder cell line for hESCs and for iPSCs. Consent was also obtained to generate iPSCs from this cell line. NclFed1A is available to the research and clinical community.

## Conclusions

To the best of our knowledge, no consistent reports exist on the derivation of new hESC lines by using feeder-free systems; therefore, the next generation of GMP-grade hESCs will require feeder cells. NclFed1A was derived because of a lack of readily available GMP-grade fibroblasts that have been consented for and validated to support hESC and iPSC culture. We showed that NclFed1A was comparable to non-GMP-grade fibroblasts widely used to support hESC derivation and culture. This cell line is available to the research and clinical community.

## Abbreviations

FBS: fetal bovine serum; FDA: Food and Drug Administration; GMP: good manufacturing practice; hESC: human embryonic stem cell; HFEA: Human Fertility and Embryo Authority; HTA: Human Tissue Authority; iPSC: induced pluripotent stem cell; KOSR: KnockOut serum replacement; KOSR-XF: KnockOut serum replacement xeno-free; MCB: master cell bank; mEFs: mouse embryonic fibroblasts; MHRA: Medicines and Healthcare Products Regulatory Agency; Neu5Gc: N-glycolylneuraminic acid; NclFed1a: CPR/HGP/Ncl/Fed1A; P: passage; PDs: population doublings from the MCB; PSB: preseed bank.

## Competing interests

The authors declare that they have no competing interests.

## Authors' contributions

MH, AM, and NP conceived and designed the study; LF derived the MCB; AB and HL established and executed the Quality Management System; NP, RS, RB, RO, GL, LF, LY, and LH characterized and qualified the cell line; and NP, MH, and AM prepared the manuscript. All authors read and approved the manuscript.

## Supplementary Material

Additional file 1 **Figure S1**. Images of NclFed1A showing **(a) **about 15% confluent, **(b) **about 60% confluent, and **(c) **100% confluent. **(d) **The number of cells per square centimeter in a confluent flask was determined by counting cells with a Vi-Cell (Beckman Coulter), in eight different confluent T150 flasks. Counts were repeated 3 times for each flask. Click here for file

Additional file 2 **Table S1**. PCR primers used to determine the presence of transcripts in NclFed1A that are predicted to be supportive of hESC cultures. **Table S2**. STR analysis with genotype copy number for allele 1 and 2. **Table S3**. The expression of pluripotency markers determined by FACs analysis after culture of four hESC lines for five passages on four different fibroblasts lines. **Table S4**. The expression of pluripotency markers determined by FACs analysis after culture of hESC lines for five passages on NclFed1A at P10, P15, P20, and P25. Click here for file
