# Supplementary figures and images for: Production and validation of a good manufacturing practice grade human fibroblast line for supporting human embryonic stem cell derivation and culture
Source: Stem Cell Res Ther. 2012 Mar 28;3(2):12. doi: 10.1186/scrt103 (PMC3392772; doi:10.1186/scrt103)

Supp Fig 1

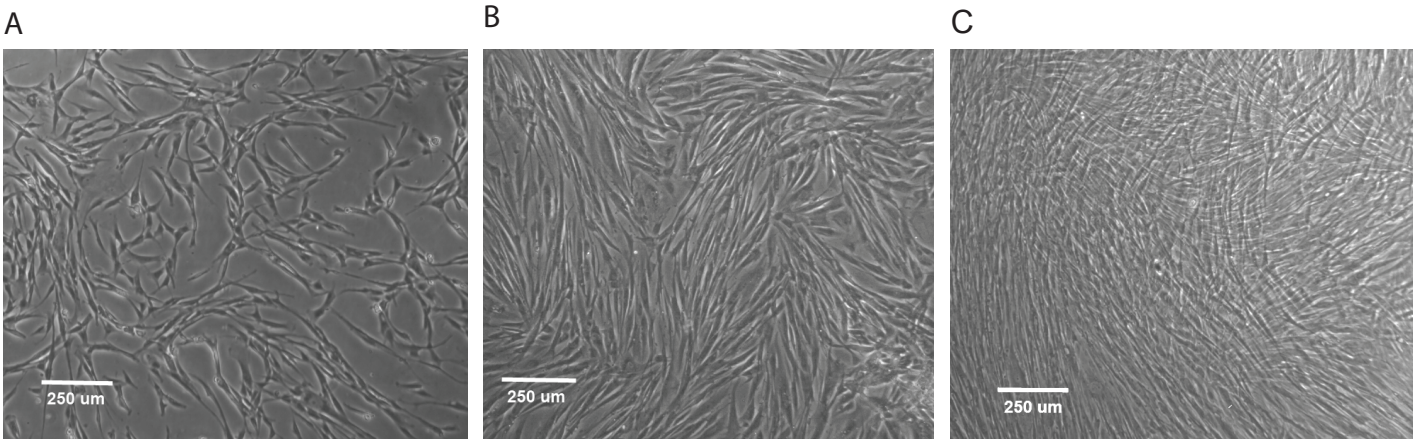

D

|                       | Mean No. of cells  | St Deviation      |
|-----------------------|--------------------|-------------------|
| Cells in a T150       | $31.9 \times 10^6$ | $1.6 \times 10^6$ |
| Cells/cm <sup>2</sup> | $2.1 \times 10^5$  | $0.1 \times 10^5$ |

Supplement: Additional file 1 — Figure S1. Images of NclFed1A showing (a) about 15% confluent, (b) about 60% confluent, and (c) 100% confluent. (d) The number of cells per square centimeter in a confluent flask was determined by counting cells with a Vi-Cell (Beckman Coulter), in eight different confluent T150 flasks. Counts were repeated 3 times for each flask. [file scrt103-S1.PDF]
