# Supplementary material for: Production and validation of a good manufacturing practice grade human fibroblast line for supporting human embryonic stem cell derivation and culture
Source: Stem Cell Res Ther. 2012 Mar 28;3(2):12. doi: 10.1186/scrt103 (PMC3392772; doi:10.1186/scrt103)
Supplement: Additional file 2 — Table S1. PCR primers used to determine the presence of transcripts in NclFed1A that are predicted to be supportive of hESC cultures. Table S2. STR analysis with genotype copy number for allele 1 and 2. Table S3. The expression of pluripotency markers determined by FACs analysis after culture of four hESC lines for five passages on four different fibroblasts lines. Table S4. The expression of pluripotency markers determined by FACs analysis after culture of hESC lines for five passages on NclFed1A at P10, P15, P20, and P25. [file scrt103-S2.DOCX]

Suppl Table 1

| **Primer** | **Forward** | **Reverse** |
| --- | --- | --- |
| **GAPDH** | GAGTCAACGGATTTGGTCGT | TTGATTTTGGAGGGATCTCG |
| **COL1A1** | GGCCCAGAAGAACTGGTACA | GCTGTTCTTGCAGTGGTAG |
| **COL3A1** | GACCCTAACCAAGGATGCAA | GGAAGTTCAGGATTGCCGTA |
| **COL5A1** | ATGAAGGTCCCAGAGGCTTT | TGCACCAGGGTTTCCTATTC |
| **b-FGF** | ATCAAAGGAGTGTGTGCTAACC | ACTGCCCAGTTCGTTTCAGTG |
| **FN1** | GAAGGCTTGAACCAACCTACG | TGATTCAGACATTCGTTCCCAC |
| **HSPG2** | TTGACCAACCCGATGACTTCA- | AAGGAGTCGTGGACGAGGAA |
| **HAS2** | TCCCTAGAAACCCCCATAAAGTT | AGGGTAGGTTAGCCTTTTCACA |
| **IGFBP3** | CCTGCCGTAGAGAAATGGAA | AGGCTGCCCATACTTATCCA |
| **ADAM33** | TCACAGCTATGGGCTGGAG | GTCCATCCAGGACCCAGTG |
| **GREM1** | AACAGTCGCACCATCATCAA | CGATGGATATGCAACGACAC' |

Supp Table 2

| **Marker** | **Genotype (Repeat No.)** | |
| --- | --- | --- |
|  | **Allele 1** | **Allele 2** |
| **D8S1179** | 13 | 14 |
| **D21S11** | 29 | 29 |
| **D7S820** | 9 | 11 |
| **CSF1PO** | 10 | 12 |
| **D3S1358** | 15 | 16 |
| **TH01** | 6 | 6 |
| **D13S317** | 11 | 13 |
| **D16S539** | 11 | 11 |
| **D2S1338** | 20 | 20 |
| **D19S433** | 14 | 14.2 |
| **VWA** | 16 | 17 |
| **TPOX** | 8 | 10 |
| **D18S51** | 13 | 17 |
| **D5S818** | 11 | 12 |
| **FGA** | 20 | 21 |

Supp Table 3

|  | **Tra-1-60** | **Tra-1-81** | **Tra-2-54** | **SSEA3** | **SSEA4** |
| --- | --- | --- | --- | --- | --- |
| **P0** |  |  |  |  |  |
| HUES-9 | 88.7 | 90.6 | 99.5 | 85.4 | 90.8 |
| RH-5 | 91.3 | 92.4 | 98.5 | 86.7 | 88.9 |
| SHEF-1 | 89.4 | 90.9 | 99.5 | 83.8 | 84.5 |
| NCL-5 | 90.4 | 91.8 | 99.8 | 87.6 | 95.6 |
| **NclFed1A** |  |  |  |  |  |
| HUES-9 | 88.5 | 91.8 | 99.4 | 87.2 | 93.8 |
| RH-5 | 91.8 | 90.6 | 98.9 | 89.2 | 93.4 |
| SHEF-1 | 90.4 | 92.2 | 100.0 | 94.4 | 94.9 |
| NCL-5 | 89.6 | 89.5 | 99.0 | 89.5 | 95.7 |
| **iMEF** |  |  |  |  |  |
| HUES-9 | 87.9 | 91.7 | 99.2 | 85.6 | 89.9 |
| RH-5 | 89.6 | 92.1 | 98.2 | 87.5 | 91.7 |
| SHEF-1 | 92.0 | 92.1 | 99.9 | 88.2 | 99.4 |
| NCL-5 | 91.1 | 90.3 | 99.1 | 88.4 | 96.3 |
| i**HDFn** |  |  |  |  |  |
| HUES-9 | 91.9 | 92.6 | 100.0 | 90.9 | 93.1 |
| RH-5 | 92.5 | 93.5 | 99.1 | 91.0 | 92.3 |
| SHEF-1 | 90.6 | 92.4 | 99.7 | 88.7 | 93.3 |
| NCL-5 | 91.8 | 91.7 | 100.0 | 89.2 | 96.2 |
| **iMRC-5** |  |  |  |  |  |
| HUES-9 | 87.3 | 88.5 | 97.4 | 86.1 | 90.6 |
| RH-5 | 88.4 | 88.7 | 97.6 | 86.4 | 89.3 |
| SHEF-1 | 85.7 | 91.1 | 100.0 | 87.9 | 91.8 |
| NCL-5 | 88.7 | 86.4 | 98.2 | 86.3 | 94.8 |

Supp Table 4

|  | **Tra-1-60** | **Tra-1-81** | **Tra-2-54** | **SSEA3** | **SSEA4** |
| --- | --- | --- | --- | --- | --- |
| **HUES-9** |  |  |  |  |  |
| NclFed1A p10 | 88.5 | 91.8 | 99.4 | 87.2 | 93.8 |
| NclFed1A p15 | 89.1 | 92.0 | 99.1 | 87.4 | 92.5 |
| NclFed1A p20 | 88.2 | 90.7 | 98.6 | 86.9 | 92.2 |
| NclFed1A p25 | 86.8 | 90.3 | 98.5 | 85.6 | 88.7 |
| **RH-5** |  |  |  |  |  |
| NclFed1A p10 | 91.8 | 90.6 | 98.9 | 89.2 | 93.4 |
| NclFed1A p15 | 91.2 | 90.7 | 98.5 | 88.6 | 94.0 |
| NclFed1A p20 | 91.4 | 89.3 | 98.4 | 88.1 | 92.1 |
| NclFed1A p25 | 89.6 | 88.5 | 97.8 | 86.8 | 89.6 |
| **SHEF-1** |  |  |  |  |  |
| NclFed1A p10 | 90.4 | 92.2 | 100.0 | 94.4 | 94.9 |
| NclFed1A p15 | 91.3 | 89.3 | 98.3 | 93.8 | 92.3 |
| NclFed1A p20 | 88.9 | 88.9 | 99.1 | 90.8 | 92.4 |
| NclFed1A p25 | 86.8 | 87.6 | 92.1 | 87.4 | 89.9 |
| **NCL-5** |  |  |  |  |  |
| NclFed1A p10 | 89.6 | 89.5 | 99.0 | 89.5 | 95.7 |
| NclFed1A p15 | 91.5 | 89.8 | 99.3 | 88.9 | 96.1 |
| NclFed1A p20 | 90.3 | 88.2 | 98.7 | 89.1 | 95.4 |
| NclFed1A p25 | 89.1 | 88.6 | 98.4 | 87.4 | 93.2 |
